# Salivary iodide status as a measure of whole body iodine homoeostasis?

**DOI:** 10.1017/S000711452400031X

**Published:** 2024-05-28

**Authors:** Eatedal Eenizan Alsaeedi, Peter Rose, Simon J. M. Welham

**Affiliations:** 1 University of Nottingham, School of Biosciences, Division of Food, Nutrition & Dietetics, Loughborough, Leicestershire LE12 5RD, UK; 2 University of Hafr Al Batin, College of Applied Medical Sciences, Division of Clinical Nutrition, Hafr Al Batin, Saudi Arabia

**Keywords:** Iodine, Iodide, Salivary iodide concentration, Urinary iodide concentration, Urinary iodide excretion, Dietary iodine intake

## Abstract

Iodine is a trace element required to produce the thyroid hormones, which are critical for development, growth and metabolism. To ensure appropriate population iodine nutrition, convenient and accurate methods of monitoring are necessary. Current methods for determining iodine status either involve a significant participant burden or are subject to considerable intra-individual variation. The continuous secretion of iodide in saliva potentially permits its use as a convenient, non-invasive assessment of status in populations. To assess its likely effectiveness, we reviewed studies analysing the association between salivary iodide concentration (SIC) and dietary iodine intake, urinary iodide concentration (UIC) and/or 24-h urinary iodide excretion (UIE). Eight studies conducted in different countries met the inclusion criteria, including data for 921 subjects: 702 healthy participants and 219 with health conditions. SIC correlated positively with UIC and/or UIE in four studies, with the strength of relationship ranging from *r* = 0·19 to *r* = 0·90 depending on sampling protocol, age, and if salivary values were corrected for protein concentration. Additionally, SIC positively correlated with dietary intake, being strongest when saliva was collected after dinner. SIC varied with external factors, including thyroid function, use of some medications, smoking and overall health status. Evidence provided here supports the use of SIC as a viable, low-burden method for determining iodine status in populations. However, small sample sizes and high variability indicates the need for more extensive analyses across age groups, ethnicities, disease states and dietary groups to clarify the relative accuracy and reliability in each case and standardise procedure.

## Graphical abstract

**Figure:**
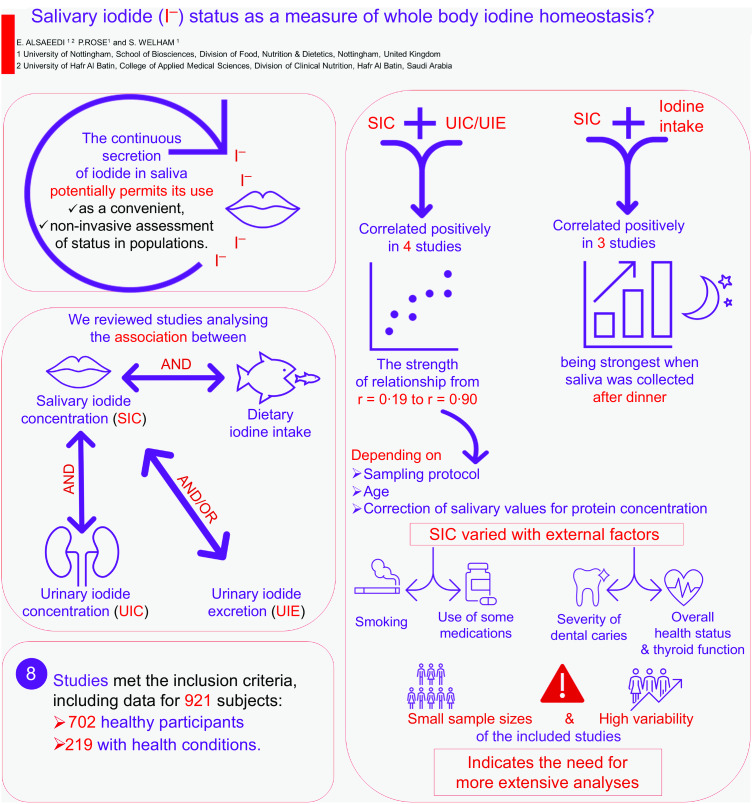

Iodine is a trace element required for the production of thyroid hormones, which are important for growth and metabolism. Since iodine is obtained from exogenous/dietary sources, it is considered an essential micronutrient, with a daily recommended intake of 120 μg d^−1^ for school-aged children (6–12 years) and 150 μg d^−1^ for adolescents and adults^(1)^. Both iodine deficiency and excess can have negative consequences resulting in a range of pathologies which impact thyroid function^(2,3)^. Iodine intake and status, unlike most other important dietary elements, are intimately linked to local geography as low concentrations in water and soil result in iodine deficiency in humans and livestock^(4)^.

The iodine content of most food sources is typically low, varies widely depending on the origins of the food and will result in low iodine intakes without parallel consumption of iodine-rich foods^(5,6)^. Most seafood is rich in iodine but generally provides a limited contribution to dietary consumption unless ingested regularly^(7,8)^. Indeed, seaweed iodine concentrations are typically high^(9)^, and people consuming it may consequently have excessive intake^(10,11)^. Moreover, drinking water that has been treated with iodine or water from aquifers in regions with high geological levels of this element can provide additional iodine^(12,13)^.

In many countries, iodine-fortified salt used for cooking remains a major source of iodine^(11,14,15)^. In addition to iodised salt, milk and dairy products are also important sources of iodine for many^(5,16,17)^, and this is particularly so in the UK, where there is no mandatory iodisation of salt^(18)^.

A key challenge to ensuring appropriate population iodine nutrition is the requirement for convenient, accurate and reliable indicators of iodine status. The median urinary iodide concentration (UIC) has long been used as a biomarker for iodine intake in the general population^(11)^, the gold standard for which is the assessment of the 24-h UIC or urinary iodide excretion (UIE). This method, however, is logistically challenging, inconvenient and time-consuming. Furthermore, due to significant intra-individual heterogeneity, it cannot be utilised reliably in individuals^(19)^. Other potential markers include serum iodide, thyroglobulin (Tg), thyroid-stimulating hormone (TSH), free triiodothyronine (FT3) and free thyroxine (FT4). These blood markers have a complementary role in iodine nutrition monitoring^(20,21)^; however, the collection of venous blood is costly and time-consuming for large-scale investigations. A potential alternative marker may be saliva, as it contains easily measurable iodide concentrations^(22,23)^ due to the transport activity of the sodium iodide symporter in salivary glands (NIS or SLC5A5)^(23)^. A key challenge to ensuring appropriate population iodine nutrition is the requirement for convenient, accurate and reliable indicators of iodine status. The median UIC has long been used as a biomarker for iodine intake in the general population^(11)^, the gold standard for which is the assessment of the 24-h UIC or UIE. This method, however, is logistically challenging, inconvenient and time-consuming. Furthermore, due to significant intra-individual heterogeneity, it cannot be utilised reliably in individuals^(19)^. Other potential markers include serum iodide, Tg, TSH, FT3 and FT4. These blood markers have a complementary role in iodine nutrition monitoring^(20,21)^; however, the collection of venous blood is costly and time-consuming for large-scale investigations. A potential alternative marker may be saliva, as it contains easily measurable iodide concentrations^(22,23)^ due to the transport activity of the NIS or SLC5A5^(23–26)^. Saliva sample collection is a convenient, more sanitary and rapid procedure than 24-h urine collection or blood-based biochemical analysis, and previous work has indicated that the salivary iodide concentration (SIC) and UIC are potentially directly related^(27)^.

We hypothesised that the concentration of iodide in saliva is positively correlated with UIC and dietary iodine intake. As such, saliva has the potential to be a viable alternative biomarker for determining iodine nutritional status across populations. We therefore conducted a scoping review to systematically map the research conducted in this field and highlight any knowledge gaps that may already exist. The following research questions were addressed:

- What are the associations between SIC and dietary iodine intake, SIC and UIC, or SIC and 24-h UIE in different population groups?
- Do the associations between SIC and dietary iodine intake significantly differ from the associations between UIC and/or 24-h UIE and dietary iodine intake?
- What is the best time to sample saliva?

## Methodology

### Search strategy

This review was performed according to the most recent checklist of the Preferred Reporting Items for Systematic Reviews and Meta-Analyses Extension for Scoping Reviews (PRISMA-ScR)^(28)^. A search strategy was developed to identify studies that analysed the relationship between SIC, dietary iodine intake, and UIC and/or 24-h UIE in different populations to evaluate whether an iodide saliva measurement could represent an appropriate biomarker of iodine status. We additionally included studies which measured diurnal SIC fluctuations and those comparing SIC by health state.

Relevant literature was identified by searching the PubMed, ScienceDirect and Web of Science databases up to 31 August 2023. The terms used during the search process were ‘dietary iodine intake’, ‘salivary iodide concentration’, ‘salivary iodine concentration’, ‘salivary iodide secretion’, ‘salivary iodine secretion’, ‘salivary iodine’, ‘circulating iodide’, ‘circulating iodine’, ‘iodine in saliva’, ‘iodide in saliva’, ‘urinary iodide’, ‘urinary iodine’, ‘urinary iodine concentration’, ‘urinary iodine excretion’, ‘iodine reabsorption’, ‘iodide reabsorption’, ‘iodide accumulation’, ‘iodine accumulation’, ‘iodine in blood’, ‘iodine in the circulation’, ‘iodine in the urine’, ‘iodine level in blood’ and ‘iodine conservation’ linked with Boolean search operators (AND, OR and NOT). The reference lists of original and review articles were also searched to identify relevant articles. The Mendeley reference manager application was used to manage all references and remove duplicates.

### Inclusion and exclusion criteria

Studies were included if they met the following criteria: (1) published in the English language; (2) peer-reviewed articles with access to the full text; (3) conducted in human participants regardless of age; (4) conducted in a small or large group across a specific population; (5) reported or not reported dietary iodine intake; and (6) reported SIC, UIC and/or UIE measurements via 24-h urine collection or alternative ways (timed sampling or spot). Studies were excluded if they (1) were published in a language other than English; (2) only the abstract was available; (3) were carried out on animals and (4) did not report SIC.

### Article selection and data extraction

**Fig. 1.**
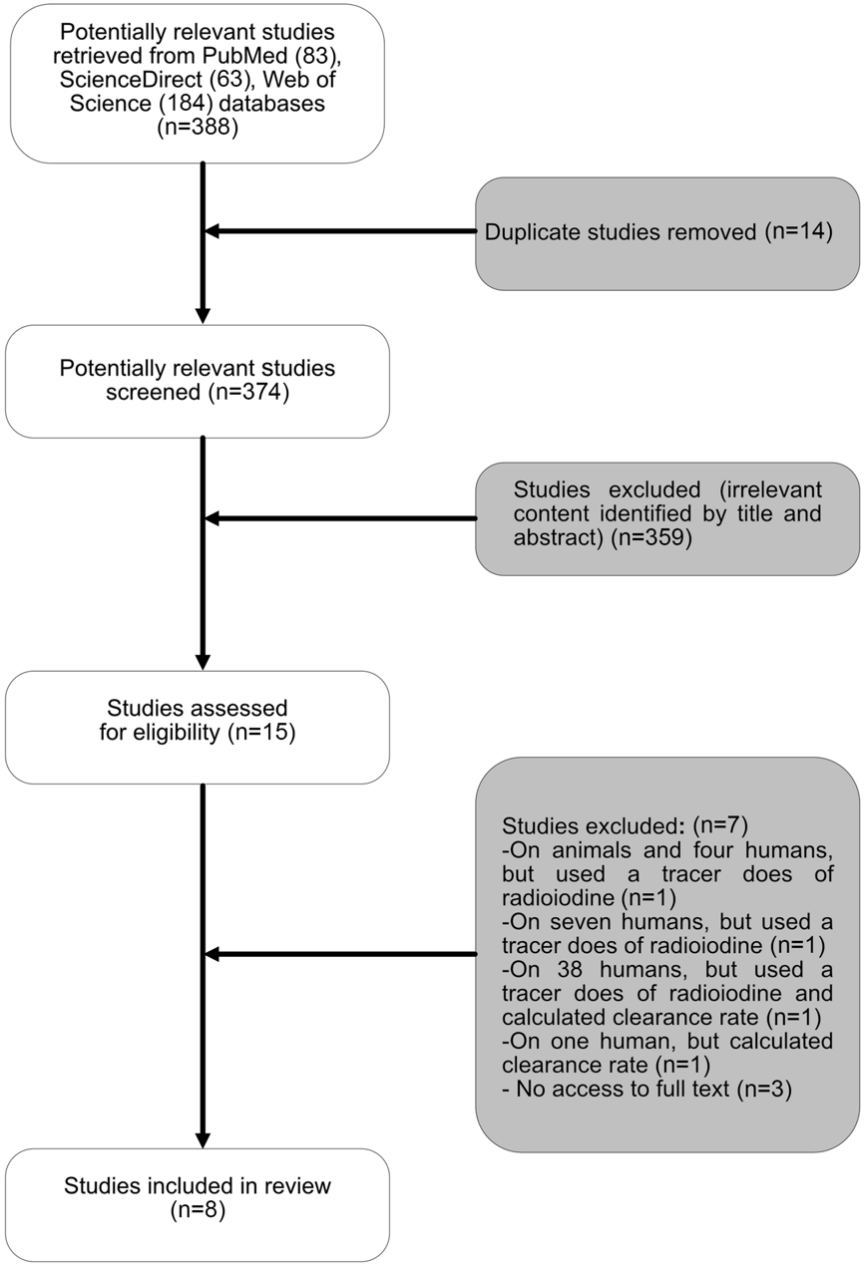
The flow chart for the process for selecting sources of evidence included in the scoping review.

Initial screening involved assessment of titles and abstracts where available by the primary author (E.A.) and subsequently rechecked by the other authors (S.W. and P.R.) to ensure accuracy. Studies meeting the eligibility criteria were selected for further evaluation via full-text analysis. Potentially relevant publications lacking an abstract were additionally assessed via full-text analysis. Additional papers were identified by reviewing the full texts. Figure 1 shows the selection process for articles.

Study data were extracted and included surname of the first author, publication year, country in which the study was conducted, study design, study period, sample size and type, participants’ age, description of the saliva and urine sampling method, the correlation coefficients between the mean or median SIC (μg l^−1^) and UIC (μg l^−1^) or UIE (μg d^−1^), correlation coefficients between SIC (μg l^−1^) and dietary iodine intake (μg d^−1^), correlation coefficients between UIC (μg l^−1^) or UIE (μg d^−1^), and dietary iodine intake (μg d^−1^). Finally, the concentration of iodide in saliva was assessed in individuals with conditions that have, or are believed to have, relevance to iodine status. These included people treated for cardiac arrhythmias with the iodinated drug amiodarone, those treated for thyroid disorders with radio iodide, those with hyperthyroidism and among smokers and people with different levels of dental caries.

### Data synthesis and statistics

The extracted data from the included studies underwent synthesis, bringing together findings within the text and tables. To ensure uniformity, units were consistently standardised, with concentrations reported in μg l^−1^ and daily intakes in μg d^−1^. Detailed information regarding correlation measurements in each included study, such as the use of Spearman’s and Pearson’s correlations, along with the consideration of a significant *P*-value, is provided in the footnotes accompanying the tables.

### ResultsStudy selection and characteristics

Fifteen studies met the inclusion criteria, of which full text was unavailable for three studies, which were consequently excluded. Four studies were additionally excluded because they used a tracer dose of radioiodine or only calculated salivary iodide clearance rate. Ultimately, eight studies were included in the final review. Five of these studies were conducted in adults^(29–33)^, two in school-aged children^(34,35)^ and one study of mixed age groups (15–60 years)^(36)^. Collectively, the eight studies (three from Europe, one from New Zealand, one from the USA, two from China and one from Turkey) included data totalling 921 subjects: 702 healthy participants, ten patients with differentiated thyroid carcinoma (DTC) on a low dietary iodine intake in order to prepare them for radioactive iodine (^131^I) therapy, thirty-eight patients with hyperthyroidism, ten patients with excessive concentrations of iodide as a result of using amiodarone for more than three months, eighty-seven smokers and seventy-four patients with different levels of dental caries (Table 1).

**Table 1.** Study characteristics

First author’s surname, year of publication and country of the study	Study design and period	Sample size (*n*) and type			Age (year)*		Study aims and method
Dekker *et al.* (2021)^(29)^ The Netherlands	Single-centre pilot study 1-d measurements	11 F and 9 M healthy individuals on normal diet			43·5	29·8–53·8	- They aimed to determine if an iodide saliva measurement accurately represents the entire iodide body pool in clinical practice by establishing the relationship between salivary iodide secretion and 24-h UIE. - The healthy participants and DTC patients did not use supplements or medications. - A polyethene swab was used to collect saliva (Salivettes, Sarstedt), and subjects were required to gently suck for 2–3 min without chewing because it causes a greater water content in saliva.
		6 F and 4 M DTC patients with a low dietary iodine intake to prepare them for ^131^I therapy			50·1	44·0–59·0	
		3 F and 7 M patients with a high iodide status due to amiodarone use for > 3 months Total = 40			71·5	51·8–75·3	
Guo *et al.* (2021)^(34)^ China	Multi-centre (randomly selected 3 boarding schools in Ningjin County) Measurements for 3 consecutive days	20 boys and 9 girls (healthy school-aged children consumed their usual diet) Total = 29			10·17	1·37	- The study aimed to confirm the correlation between SIC and iodine intake, explore the intra-individual, intra-day, and population variation in SIC values, as well as deduce the best sampling time and sample size that could be applied in epidemiological surveys to evaluate the nutritional status of iodine. - Students had preliminary screening by examining anthropometric data and having blood samples taken to check thyroid function. - The children were asked to naturally secrete saliva without any stimulus. - No significant differences were observed in the baseline characteristics of the participants.
Guo *et al.* (2020)^(35)^ China	Multi-centre (randomly selected 3 boarding schools from Ningjin County) 1-d measurements	223 boys and 200 girls (healthy school-aged children consumed their usual diet) Total = 423			10·3	1·0	- This study sought to analyse the correlation between SIC and iodine consumption, UIC, and thyroid function to assess the effectiveness of SIC as a functional biomarker for determining iodine status. - Students had preliminary screening by examining anthropometric data and having blood samples taken to check thyroid function. The weight, height, body surface area and BMI of the girls were relatively lower than those of boys (*P* < 0·05). - The children were asked to secrete saliva naturally without any stimulus.
Suciu *et al*. (2018)^(30)^ Romania	Single-centre study 1-d measurements		Non-smoker	Smoker	NA		- This preliminary study aimed to compare the salivary iodide concentrations in the control group (smoker and non-smoker) with patients (smoker and non-smoker) with different levels of dental caries. - The participants’ baseline characteristics were unavailable. - The SIC were determined using the Sandell–Kolthoff reaction.
		Control group	15	15			
		Patients with simple carious lesions	15	15			
		Patients with chronic marginal periodontitis	15	15			
		Patients with apical periodontitis pathology	15	15			
		Total = 120					
Gulaboglu *et al.* (2012)^(31)^ Turkey	Single-centre study 1-d measurements	12 F and 14 M healthy volunteers			17–26		- They looked at whether there was a link between iodide concentrations in saliva and urine in healthy volunteers and patients with dental caries. - They studied patients with advanced dental caries (≥ 5). - Unstimulated saliva was collected by having participants salivate into sterile tubes. - Participants did not use iodine-containing supplements or medication.
		12 F and 17 M patients with dental caries Total = 55			18–26		
Ford *et al.* (1997)^(32)^ New Zealand	Single-centre study 1-d measurements	93 healthy adult volunteers			NA		- Their first aim was to compare the activity or concentrations of ten constituents of saliva including iodine in patients with hyperthyroidism with normal subjects. - The participants’ baseline characteristics were unavailable. - During a 5-min interval, the participants chewed raw gum and the saliva was collected into a plastic container.
		38 adult patients with hyperthyroidism Total = 131			NA		
Tenovuo & Makinen (1976)^(36)^ Finland	1-d measurements	Non-smoker	Smoker		15–60		- This study aimed to compare the salivary iodide and thiocyanate concentrations in the smoker with non-smoker group. - Volunteers whole saliva was collected using paraffin stimulation. - The collection of saliva samples was conducted between 08.00 and 15.00. - The SIC were measured electrometrically with an iodine ion electrode.
		64 F	16 F				
		28 M	11 M Total = 119				
Vought & London (1965)^(33)^ USA	Measurements for 10 d (5 d in a row (the day-to-day period), and on one randomly chosen day over the course of 5 weeks (the week-to-week period))	Four healthy adult females			NA		- They aimed to find the correlation of SIC to dietary iodine intake or the concentrations of plasma (or serum) inorganic iodine. - Participants from different families following their usual dietary, work and recreational habits. - The participants’ baseline characteristics were unavailable. - The participants’ whole saliva was collected using paraffin stimulation (dental utility wax).

F, females; M, males; DTC, differentiated thyroid carcinoma; ^131^I, radioactive iodine; NA, not available.

* Data are expressed as range or means and standard deviations or median with interquartile range (IQR) value depending on the study.

SIC and UIC were analysed using inductively coupled plasma mass spectrometry (ICP-MS)^(29,34,35)^, the Sandell–Kolthoff reaction method^(30,31)^, and electrometrically with an iodine sensitive ion electrode^(36)^. Data are not available about the method of measurement in two studies^(32,33)^. Six studies collected samples for 1 d^(29–32,35,36)^, one study collected samples for three consecutive days^(34)^ and one study collected for 10 d (5 d in a row (the day-to-day period), and on one randomly chosen day over the course of 5 weeks (the week-to-week period))^(33)^.

The collection methods for saliva samples varied among the studies, with all but one providing details on their sampling techniques. In one study, participants were instructed to gently suck on a swab for approximately 3 min^(29)^. Three studies required subjects to naturally secrete saliva without any external stimuli^(31,34,35)^. Another two studies collected stimulated saliva samples using paraffin stimulation (dental utility wax)^(33,36)^, for exactly 4 min^(33)^. The last study instructed participants to chew raw gum and collect samples into a plastic container over a 5-min period^(32)^. Details about the eight studies are provided in Table 1.

In addition to the variation in collection methods, the number of collected samples differed. Saliva samples were collected six times a day (prior to and after the three main meals) over three consecutive days in a recent study by Guo *et al*. (2021)^(34)^, 2 h following breakfast in Gulaboglu *et al*.’s (2021) study^(31)^, at the same time as the collection of 24-h urine samples without determining the number of collected samples in Guo *et al*.’s (2020) study^(35)^, 2 h after the evening meal over 10 d in Vought & London’s study^(33)^, and one time in the period between 08.00 and 15.00 in a study by Tenovuo J & Makinen (1976)^(36)^. In another study conducted by Dekker *et al*. (2021), nine saliva samples were collected at 2-h intervals from healthy volunteers, starting either at 07.00 or 08.00 and concluding at 23.00 or 24.00, with one morning sample from the patient groups^(29)^. Therefore, participants’ morning saliva samples were used in the statistical analysis (Tables 2 and 3).

**Table 2.** The relationship between SIC and UIC and/or UIE

Reference	Sample size (*n*) and type	SIC (μg/l)*		UIC (μg/l)* Or UI/Cr (μg/g)* ^,†^		24-h UIE (μg/d)*		Correlation coefficient^¶^	Sample collection and analysis methods
Dekker *et al.* (2021)^(29)^	20 healthy volunteers	136	86–308‡	102	88–141	176	96–213	SIC** and 24-h UIE (*r* = 0·80, *P* < 0·01) SIC** and UI/Cr (*r* = 0·85, *P* < 0·01) SI/SP†† and 24-h UIE (*r* = 0·90, *P* < 0·01) SI/SU†† and 24-h UIE (*r* = 0·86, *P* < 0·01)	-The SIC and UIC were measured using an ICP-MS assay. -A 24-h urine sample was collected for 1 d. -The UIE was expressed as daily urine iodine excretion (μg/d) based on 24-h urine samples and the UI/Cr (μg/gCr). -The Kruskal–Wallis test revealed that SIC, UI/Cr and UIE were significant across the three groups with a *P*-value < 0·01. -The healthy volunteers collected nine saliva samples at 2-h intervals, commencing at either 07.00 or 08.00 (sample 1) and concluding at either 23.00 or 24.00 (sample 9). -Patient groups collected one saliva sample in the morning. -Participants’ morning saliva samples were used in the statistical analysis. -Correcting for water content using salivary urea and protein correction decreased the intra-individual CV for SIC from 63·8 % to 26·9 % and 37·7 %, respectively, and the inter-individual CV from 77·5 % to 47·0 % and 41·6 %, respectively, and they were the lowest for SIC.
	10 DTC patients	72	30–95	25	15–28	26	22–37||		
	10 patients on amiodarone	14 × 10‡	(11 × 10‡–26 × 10‡)§	9 × 10‡	8 × 10‡–11 × 10‡	10 × 10‡	8 × 10‡–11 × 10‡		
Guo *et al.* (2021)^(34)^	29 healthy school-aged children	229	132–348	168	88–338	146	87–267	SIC before and after breakfast *v*. 24-h UIC (*r* = 0·19; *P* > 0·05, *r* = 0·36; *P* < 0·05, respectively) SIC before and after lunch *v*. 24-h UIC (*r* = 0·39 and 46; *P* < 0·05, respectively) SIC before and after dinner *v*. 24-h UIC (*r* = 0·65 and 0·40; *P* < 0·05, respectively) SIC before and after breakfast *v*. 24-h UIE (*r* = 0·34: *P* > 0·05, *r* = 0·52; *P* < 0·05, respectively) SIC before and after lunch *v*. 24-h UIE (*r* = 0·52 and 0·50; *P* < 0·05, respectively) SIC before and after dinner *v*. 24-h UIE (*r* = 0·59 and 46; *P* < 0·05, respectively)	-The SIC and UIC were measured using an ICP-MS assay. -The UIE was calculated for everyone by multiplying UIC (μg/l) by total urine volume (litter). -A 24-h urine and saliva samples were collected for three consecutive days. -Saliva samples were collected six times a day (prior to and post of the three main meals).
Guo *et al.* (2020)^(35)^	423 healthy school-aged children	106	68–181	121 (76–199) + SUIC (μg/l)* 124 (64–231)		90	57–136	SIC and 24-h UIC (*r* = 0·37, *P* < 0·0001) SIC and 24-h UIE (*r* = 0·40, *P* < 0·0001) SIC and SUIC (*r* = 0·27, *P* < 0·0001)	-The SIC, SUI and UIC were measured using an ICP-MS assay. -The spot urine was obtained before the collection of 24-h urine. -The saliva and 24-h urine samples were collected at the same time. -The UIE was calculated for everyone by multiplying UIC (μg/l) by total urine volume (litter). -There were no significant relationships between SIC and serum iodine or thyroid function indicators (FT3, FT4 and TSH; *P* > 0·05).
Gulaboglu *et al.* (2012)^(31)^	26 healthy volunteers	83	28	120	32	NA		SIC and SUIC (*r* = 0·519, *P* < 0·001)	-All subjects were instructed to refrain from eating, drinking or using breath fresheners for a minimum of 2 h before the saliva, and plaque samples were collected following breakfast and the urine samples were also collected at the same time. -The SUIC and SIC were determined using the Sandell–Kolthoff reaction. -The urinary and salivary iodide concentrations for healthy subjects were higher than that for patients with dental caries (*P* < 0·0001). -There was a positive correlation between the iodide concentrations in saliva and urine for both healthy people and patients with dental caries. -There was no significant difference in iodide concentration (saliva and urine) for patients with dental caries and normal subjects, as well as between males and females (no available data).
	29 patients with dental caries	44	22	75	27			SIC and SUIC (*r* = 0·750, *P* < 0·001)	

SIC, salivary iodide concentration; UIC, urinary iodide concentration; UIE, urine iodide excretion; DTC, differentiated thyroid carcinoma; UI/Cr, urinary iodide:creatinine ratio; SI/SP, salivary iodide:protein ratio; SI/SU, salivary iodide:urea ratio; ICP-MS, inductively coupled plasma mass spectrometer; SUIC, spot urinary iodide concentration; FT3, free triiodothyronine; FT4, free thyroxine; TSH, thyroid-stimulating hormone; NA, not available.

* Data are expressed as means and standard deviation or median with interquartile range (IQR) value depending on the study.

† UI/Cr.

‡ Two samples were missing: hence, *n* 18.

§ One sample was missing: hence, *n* 9.

|| One DTC patient had a total 24-h urine volume < 600 ml: hence, *n* 9.

¶ Spearman’s correlation was used to establish the correlations between the variables. *P* < 0·05 was considered significant; except in Gulaboglu *et al.*’s study (2012), Pearson’s correlation coefficient was used.

** There were four missing samples: hence, *n* 36.

†† There were twelve missing samples: hence, *n* 28.

**Table 3.** The relationship between SIC *v.* dietary intake of iodine compared with UIC or UIE *v.* dietary intake of iodine

Reference	Sample size (n) and type	Dietary intake of iodine (μg/d)*		SIC (μg/l)*		Correlation coefficient†	Sample and iodine intake collection and analysis methods
Guo et al. (2021)^(34)^	29 healthy school-aged children	258	135–433	229	132–348	SIC before and after breakfast *v.* iodine intake (*r* = 0·19; *P* > 0·05, *r* = 0·39; *P* < 0·05, respectively) SIC before and after lunch *v.* iodine intake (*r* = 0·41 and 46; *P* < 0·05, respectively) SIC before and after dinner *v.* iodine intake (*r* = 0·64 and 0·68; *P* < 0·05, respectively) 24-h UIE and iodine intake (*r* = 0·67, *P* < 0·0001) 24-h UIC and iodine intake (*r* = 0·61, *P* < 0·0001)	-Students had ate all their meals together and used an iodine-free plastic cups for drinking. -Researchers weighed and documented the food quantity both before and after each meal. -Students reported their snack intake, which was similarly recorded in the overall dietary intake. -The concentration of iodide in saliva, water, food and urine was measured using an ICP-MS assay. -The 24-h UIE and faeces iodide excretion made up the total daily iodide excretion. -The UIE was calculated for everyone by multiplying UIC (μg/l) by total urine volume (litter). -Saliva samples were collected six times a day (prior to and post of the three main meals). -There were strong positive correlations between SIC and iodine intake, particularly for the saliva samples taken before and after dinner. -When compared with 24-h UIC and UIE, saliva collected after dinner had the strongest correlation with iodine intake (0·61, 0·67, *r* = 0·68; *P* < 0·0001, respectively).
Guo *et al.* (2020)^(35)^	423 healthy school-aged children	97	62–148	106	68–181	SIC and iodine intake (*r* = 0·40, *P* < 0·0001)	-They estimated iodine intake using a special formula‡. -A 10-ml sample of drinking water was collected using an iodine-free polyethylene plastic bottle. -The iodide concentrations of drinking water and saliva were measured using an ICP-MS assay.
Vought & London (1965)^(33)^	Four healthy females	NA		NA		SIC and iodine intake (*r* = 0·937) UIC and iodine intake (*r* = 0·764) UIE and iodine intake (*r* = 0·709)	-Samples were collected from everyone 1 to 2 h after the evening meal. -Participants’ meals were stored in their homes’ refrigerators and collected daily by a staff member. The collected meals underwent processing through homogenisation and lyophilisation methods. -The only dietary modification mandated by the study was the exclusion of iodised salt from the table and kitchen. -Using the Zak method modified by Benotti, chemical iodine analyses were carried out. -Dietary iodine intake and SIC showed a strong association, while the relationship between dietary iodine intake and UIE was relatively lower.

SIC, salivary iodide concentration; UIC, urinary iodide concentration; UIE, urine iodide excretion; ICP-MS, inductively coupled plasma mass spectrometer assay; NA, not available.

* Data are expressed as means and standard deviations or median with interquartile range (IQR) value depending on the study.

† Spearman’s correlation was used to establish the correlations between the variables. *P* < 0·05 was considered significant.

‡ Daily iodine intake = UIC (μg/l) ÷ 0·92 × (0·0009 l/h/kg × 24-h/d) × weight (kg).

Dietary iodine intakes were assessed in three studies using various methods. In one study, participants’ meals were stored in their home refrigerators and collected daily by a staff member. The collected meals underwent processing through homogenisation and lyophilisation^(33)^. The only dietary modification required in this study was the exclusion of iodised salt from the table and kitchen. In another study, involving children, a special formula based on the UIC and body weight was used to estimate iodine intake. A 10-ml sample of drinking water was collected using an iodine-free polyethylene plastic bottle, and the concentration of iodide was measured using ICP-MS^(35)^. A second study with children required collective dining for all meals^(34)^. Researchers weighed and documented the food quantity both before and after each meal. Also, students reported their snack intake, which was similarly recorded in the overall dietary intake (Table 3).

Values for dietary iodine intake ranged from 97 μg d^−1^ to 258 μg d^−1^. UIC and UIE ranged from 25 μg l^−1^ to 9 × 10^3^ μg l^−1^ and 26 μg d^−1^ to 10 × 10^3^ μg d^−1^, respectively. While variation in SIC ranged from 20 μg l^−1^ to 14 × 10^3^ μg l^−1^.

### Study findings

#### The relationship between salivary iodide concentration and urinary iodide concentration and/or urinary iodide excretion

SIC were found to correlate positively with UIC and/or UIE in the four studies which measured both parameters (Table 2), with the strength of relationship ranging from *r* = 0·19 to *r* = 0·90 depending on sampling protocol, age and if salivary values were corrected for protein concentration. The tightest relationship was seen when 24-h UIE was calculated and associated with saliva collected for 3 min using chewable swabs and corrected for salivary protein^(29)^.

The strength of relationship between UIC and SIC was greater for adult groups^(29,31)^ than for children^(34,35)^. However, in all cases where diet was the only iodine source (i.e. excluding the amiodarone treated group in one study^(29)^), the association between SIC and UIC was generally much stronger for those with urinary concentrations indicative of deficiency (*r* = 0·75 – *r* = 0·90; < 100 μg l^−1^) than for those who appeared replete (*r* = 0·19 – *r* = 0·65).

SIC was typically expressed as standalone concentrations, often with correction for urea and protein concentrations (SI/SU (μg mM^−1^) and SI/SP (μg g^−1^), respectively). These steps were used to control for differences in water content from inadvertent stimulation of the salivary glands. After correcting for salivary urea and/or protein, the associations observed were even stronger (SI/SU and 24-h UIE, *r* = 0·86, *P* < 0·01, SI/SP and 24-h UIE, *r* = 0·90, *P* < 0·01^(29)^), and intra-individual (CV for SIC decreased from 63·8 % to 26·9 % and 37·7 %, while inter-individual CV decreased from 77·5 % to 47·0 % and 41·6 %, respectively. Values for CV after correction were also lowest for SIC measurements^(29)^.

In addition to variations in the strength of the relationship based on age groups, UIC and correction of saliva samples for protein and urea, the timing of saliva collecting prior to or post the three main meals also played a significant role in shaping the associations between SIC and UIC and/or UIE. A recent study found intriguing patterns in these associations^(34)^, specifically that SIC sampled before and after breakfast showed weak and modest correlations with 24-h UIC (*r* = 0·19, *P* > 0·05, and *r* = 0·36, *P* < 0·05, respectively). In contrast, SIC collected before and after lunch demonstrated stronger correlations with 24-h UIC (*r* = 0·39, *P* < 0·05, and *r* = 0·46, *P* < 0·05, respectively). The most notable findings were observed in the evening, with SIC before dinner displaying the highest correlations with 24-h UIC (*r* = 0·65, *P* < 0·05). These studies reported similar trends in the associations between SIC before and after meals and 24-h UIE, with evening measurements showing the most robust correlations (*r* = 0·59, *P* < 0·05). These findings suggest that the relationship between SIC and UIC and/or UIE is likely influenced by meal timing, with evening measurements exhibiting the strongest correlation. These findings emphasise the significance of considering meal-related fluctuations when assessing the reliability of SIC as an indicator of iodine status.

#### The effectiveness of salivary iodide concentration compared with urinary iodide concentration or urinary iodide excretion as a predictor of dietary iodine intake

The concentrations of iodide in saliva were found to correlate positively with dietary iodine intake in three studies (Table 3). These results were compared with aid correlations between UIC or UIE and dietary iodine intake in the same population group. This comparison was used to assess the accuracy of SIC compared with that of UIC or UIE in reflecting iodine status. Among the studies that assessed the relationships, almost all participants (452 out of 456) were children^(34,35)^, with values for only four adults^(33)^.

In the research of Guo *et al*., conducted on 29 healthy school-aged children^(34)^, salivary samples were collected before and after the three main meals over three consecutive days. The strength of correlation between SIC and iodine intake increased over the course of the day with the greatest degree of association being before (*r* = 0·64, *P* < 0·05) and after dinner (*r* = 0·68, *P* < 0·05). When considering the associations between UIC or UIE *v*. dietary iodine intake, they also reported similar correlations between 24-h UIE and iodine intake (*r* = 0·67, *P* < 0·0001), as well as between 24-h UIC and iodine intake (*r* = 0·61, *P* < 0·0001).

In one study on adult participants^(33)^, four healthy females collected saliva samples 1–2 h after dinner for 10 d. Similar to the findings of Guo *et al*.^(34)^, the study highlighted a strong and positive correlation between SIC and dietary iodine intake (*r* = 0·937, *P*-value = not available) when saliva was sampled during the evening. Furthermore, consistent with these results, strong associations were identified between UIC and iodine intake (*r* = 0·764, *P*-value not available) and between UIE and iodine intake (*r* = 0·709, *P*-value not available)^(33)^. Single samples taken in a larger cohort of children (423) revealed a much weaker, albeit still significant, association between SIC and iodine intake (*r* = 0·40, *P* < 0·0001). However, it is unclear when saliva samples were taken, and if the correlation between UIC or UIE *v*. iodine intake was significantly different^(35)^.

#### The daily variation in salivary iodide concentration is lower than that of urinary iodide concentration and urinary iodide excretion

Recent nutritional iodine intakes may contribute to the variation in salivary iodide and increase variation in concentrations both across the day and subsequent days. Recent study measured the variation in iodide concentrations in saliva at different times of the day (before and after the three main meals) over 3 d and showed that the mean CV was lowest (67·39 %) before breakfast, increasing later in the day, with the greatest variation in SIC occurred post-dinner, with a mean CV of 104·40 %. Additionally, the researchers observed that the SIC variation was lowest at the person level^(34)^. The mean CV within individuals for SIC was 42·73 %, which was less than that of iodine intake (53·12 %), 24-h UIE (49·69 %) and 24-h UIC (47·71 %), respectively. Moreover, the variation in SIC was lower at the population level (71·29 %) than 24-h UIE (72·49 %) and 24-h UIC (100·43 %). By comparison, the mean CV of iodine intake (66·70 %) was somewhat less than that of SIC, UIC or UIE^(34)^.

#### Salivary iodide concentration can vary depending on several factors and is associated with specific health challenges

Iodide concentration in saliva depending on factors like an individual’s iodine intake, thyroid function, use of some medications like potassium iodide, smoking and overall health status. One study reported the effect of a type of medication (amiodarone) and a low-iodine diet (LID) (< 50 μg d^1^) on SIC, two studies reported the effect of smoking on SIC, while three studies considered the difference between individuals with different health issues and those who are in good health and are summarised in Table 4.

**Table 4.** Comparison of the SIC in healthy people with those with different statuses

Reference	Status	Sample size (*n*) and type			SIC (μg/l)*		Notes
Dekker et al. (2021)^(29)^	Using some medications	20 healthy volunteers			136 (86–308)†		The salivary iodide concentrations for patients on amiodarone was higher than that for normal subjects.
		10 patients on amiodarone			14 × 10 (11 × 10–26 × 10)		
Dekker et al. (2021)^(29)^	Intake status	20 healthy volunteers			136 (86–308)†		The salivary iodide concentrations for patients with DTC on a very low iodine diet was lower than that for normal subjects.
		10 DTC patients on a VLID			72 (30–95)		
Suciu *et al*. (2018)^(30)^	Smoking		Non-smoker	Smoker	Non-smoker	Smoker	-Smoker persons had lower SIC than non-smokers.
		Healthy volunteers	15	15	97	20	
		Patients with simple carious lesions	15	15	295	120	
		Patients with chronic marginal periodontitis	15	15	279	190	
		Patients with apical periodontitis pathology	15	15	220	168	
Tenovuo & Makinen (1976)^(36)^		Healthy volunteers	92	27	1800±1100	1100±400	-Smoker persons had lower SIC than non-smokers (*P* < 0·001).
Suciu *et al*. (2018)^(30)^	Health status	15 healthy volunteers			97		-The SIC decreased with increasing severity of dental caries.
		15 patients with simple carious lesions			295		
		15 patients with chronic marginal periodontitis			279		
		15 patients with apical periodontitis pathology			220		
Gulaboglu et al. (2012)^(31)^		26 healthy volunteers			83 ± 28		The salivary iodide concentrations for patients with dental caries was lower than that for healthy subjects (*P* < 0·0001).
		29 patients with dental caries			44 ± 22		
Ford et al. (1997)^(32)^		93 healthy volunteers			1054‡#		The salivary iodide concentrations for patients with hyperthyroidism was higher than that for normal subjects (*P* > 0·05).
		38 patients with hyperthyroidism			1226§#		

SIC, salivary iodide concentration; DTC, differentiated thyroid carcinoma; VLID, very low iodine diet.

* Data are expressed as means or means and standard deviations or median or median with interquartile range (IQR) value depending on the study.

† Two samples were missing; hence, *n* 18.

‡ Five samples were missing; hence, *n* 88.

§ Four samples were missing; hence, *n* 34.

# These values have converted from µmol/l to µg/l for consistency.

Heart rhythm disorders, such as atrial fibrillation, are treated or prevented using the drug amiodarone^(37)^. A 100 mg of amiodarone tablet contains 250 times the recommended daily iodine requirement for an adult^(38)^. In individuals using amiodarone for more than 3 months (*n* 10), the median concentration of iodide in patients’ saliva was about 100 times higher than that of healthy (*n* 20) individuals (14 × 10^3^ μg l^−1^ and 136 μg l^−1^, respectively(^(29)^.

For patients scheduled for DTC radioactive iodine (^131^I) therapy, a very low iodine diet (< 50 μg d^−1^) has to be adhered to. Those patients following this diet are reported to have a much lower concentration of salivary iodide (approximately half) than that of healthy volunteers not following any specific iodine reduction diet (72 μg l^−1^ *v*. 136 μg l^−1^, respectively)^(29)^.

In addition to iodine restrictive diets, smoking is also reported to influence SIC by increasing the concentration of thiocyanate, a known competitive inhibitor of the NIS, the consequence of which is to decrease salivary iodide (non-smokers healthy volunteers had a substantially higher mean SIC (1800 ± 1100 μg l^−1^) compared with that of smokers (1100 ± 400 μg l^−1^))^(36)^. These findings further confirmed by a recent work, and researchers have shown that among healthy volunteers, patients with simple carious lesions, patients with chronic marginal periodontitis, and patients with apical periodontitis pathology, that SIC for those who smoked were lower compared with the non-smokers by 80 %, 59 %, 32 % and 24 %, respectively^(30)^.

Other SIC-modifying conditions include dental caries. In individuals with dental caries, a significant reduction in iodine status as measured by both salivary and urinary concentrations has been reported^(31)^. The authors showed that patients with dental caries had a 38 % lower SIC than healthy controls (75 ± 27 μg l^−1^ *v*. 120 ± 32 μg l^−1^, respectively), and this finding is suggestive of reduced iodine status, which was supported by diminished urinary concentrations *viz*. 47 % lower (44 ± 22 μg l^−1^ *v*. 83 ± 28 μg l^−1^ respectively; *P* < 0·001). In addition, another study found that the SIC decreased with increasing severity of dental caries^(30)^. Patients with simple carious lesions had the highest mean SIC values (295 μg l^−1^) followed by those with chronic marginal periodontitis (279 μg l^−1^) and patients with apical periodontitis pathology (220 μg l^−1^). However, the SIC in the control group was the lowest (97 μg l^−1^) when compared with the patient groups^(30)^.

Lastly, elevated iodine status as indicated by salivary concentrations was suggested to be associated with hyperthyroidism^(32)^. Participants with hyperthyroidism (*n* 38) had numerically higher median concentrations of iodide in their saliva (1226 μg l^−1^) as compared with normal subjects (*n* 93; 1054 μg l^−1^), although the measured differences were not statistically significant, likely due to the low number of participants. However, stimulated salivary flow rate was significantly increased, and this raises the possibility that total salivary iodide secretion may also be influenced.

## Discussion

Several studies aimed at the detection of SIC have been conducted^(39,40)^. Interestingly, the salivary glands, like the thyroid, have the capacity to concentrate iodide by virtue of expressing the NIS^(23)^. The epithelium of the parotid gland’s intralobular ducts is the principal place where iodide is transported into saliva^(22,41)^. The iodide is taken from the periductal capillaries, concentrated by the striated duct cells, released into the duct lumen and finally transferred into the oral cavity^(23,24)^. Its concentration in parotid saliva is higher than in gastric juice under situations of minimal stimulation^(42)^. In this scoping review, we provide the first assessment of articles seeking to establish the usefulness of salivary iodide measurement as an indicator of status. We considered papers which compared the association between SIC and more traditional measures of iodine status, in addition to those assessing the influence of specific treatments and health conditions on SIC.

From the studies considered herein, evidence would suggest that saliva could represent a viable bio-matrix to allow determination of iodine status. This offers an alternative means for the assessment of iodine nutrition status that is non-invasive and rapid and can be collected by people with little clinical experience^(43)^. Additionally, the consistency in salivary iodide secretion within individuals across multiple days lends further credence to its validity.

An important finding of this review is the positive relationship observed between SIC and UIC/UIE. This correlation was particularly strong in studies involving adults and individuals with UIC indicative of deficiency. This suggests that SIC may serve as a reliable indicator of iodine status, especially in populations at risk of iodine deficiency. Notably, the strength of the relationship was influenced by factors such as age, meal timing and the correction of SIC for salivary protein or urea concentration. In particular, evening measurements of SIC showed the strongest correlation with UIC/UIE, emphasising the importance of considering meal-related fluctuations when using SIC as an iodine biomarker. This insight highlights the need for the development of a standardised set of protocols for saliva collection, to ensure consistency in measurements and to strengthen the validity of SIC as an indicator of iodine status. Furthermore, when comparing the correlations between SIC and dietary iodine intake with UIC/UIE, iodine intakes within the same population groups revealed some interesting patterns. SIC correlated positively with dietary iodine intake, mirroring the relationships observed with UIC/UIE. These findings suggest that SIC may be as informative as UIC/UIE in reflecting iodine nutritional status, in both children and adults. However, it is essential to acknowledge that the strength of the correlations between SIC and iodine intake may vary based on factors like sampling time and age groups, and additional research is warranted.

It is also noteworthy that the specific health conditions can also impact SIC. Amiodarone use, for instance, resulted in substantially elevated SIC, highlighting the impact of medication on SIC. Moreover, individuals with DTC following a LID had significantly lower SIC, indicating the influence of health conditions and dietary restrictions on SIC. Furthermore, dental caries and hyperthyroidism were associated with altered SIC, emphasising the potential of SIC to reflect underlying health challenges.

The ability to reliably assess iodine status using salivary iodide measurement raises the possibility that it could be employed for monitoring patients under specific clinical circumstances. For instance, after complete thyroidectomy, many patients with DTC are provided with ^131^I therapy to remove any remaining thyroid cancer tissue^(44)^. A LID(< 50·0 μg/d) is required for a 1–2 weeks prior to therapy to lower the body’s iodide stores and to increase the uptake of administered ^131^I^(45)^. The status of iodine in these patients is required to determine compliance with the LID and likely dictates efficacy of the intervention. In certain situations, patients may not be eligible for immediate ^131^I treatment due to their recent exposure to diagnostic imaging involving iodinated contrast agents or the use of medications like amiodarone^(44)^, which can lead to an excess of iodide in the body. Consequently, rapid and practical measurement of the iodine body pool, such as using saliva samples, can assist clinicians to plan this therapy more effectively. The consistent observation of a strong positive relationship between SIC and UIC/UIE does support the potential utility of SIC as an indicator of iodine status in individuals. However, data are relatively limited, and the effect on SIC of variability in dietary patterns, age, illness, sex, life stage and salivary secretion control have yet to be determined, so its use for individual monitoring may still be some way off.

A lack of iodine might be one of the contributing factors in the formation of dental caries, and this could be a possible explanation for the lower concentrations of iodide in the saliva and urine of patients compared with healthy persons^(31)^. Indeed, it is compelling that decreasing SIC correlates with increasing severity of dental caries^(30)^. This link suggests that iodine, in addition to its importance for thyroid hormone synthesis, may also play a role in maintaining oral health. A research study proposed a hypothesis that highlights the potential significance of iodide in oral and salivary gland health, suggesting its trophic, antioxidant, apoptosis-inducing and assumed antitumour effects as crucial for preventing disorders in these tissues^(25)^.

Iodine-containing antimicrobials, such as the widely adopted povidone-iodine, which may be used as a mouthwash, is very effective in killing bacteria, viruses, and fungi and was recently adopted as a means of controlling severe acute respiratory syndrome coronavirus 2 (SARS-CoV-2) orally^(46)^. While it is clear that iodine is the active component responsible for the antimicrobial function, this is mediated by molecular iodine (I_2_) which can penetrate the microbial cell and oxidise a number of proteins, alongside fatty acids and nucleotides, resulting in cell death^(47)^. Salivary secretions instead release I^−^, which must be modified to another form (e.g. I_2_ or HOI) in order to confer antimicrobial function and so indicates that additional biochemical processes must occur in the mouth for the secreted iodide to have an impact. There is, nonetheless, growing evidence to support the idea that a consistent secretion of salivary iodide may act to control oral microbial populations.

The studies described here adopt a range of methods for determining iodine nutritional status. Since > 90 % of ingested iodine is eliminated in the urine, UIC is a popular measure of recent iodine consumption^(48)^. In large-scale population studies, however, collecting 24-h urine samples is challenging and impractical. Furthermore, it is difficult to be certain about the completeness of urine collection, and there is currently no agreement on the optimal strategy for quality assurance. There are methods to evaluate the completeness of urinary collection, including analysing the recovery of ingested para-aminobenzoic acid, an exogenous marker, in the urine, but this might not be suitable for large population investigations^(49)^. Total urinary volume measurement (volumes less than 300 ml per 24 h are typically regarded as low and a sign of under-collection), self-reporting of incomplete collection and evaluation of urinary creatinine excretion^(50,51)^ are also useful methods but may be prohibitive for larger studies. When utilised alone, none of these techniques provide completely reliable results^(52)^; therefore, strict procedures to reduce excessive and inadequate urine collection should be used. In this review, three studies^(29,34,35)^ collected 24-h urine samples, and only one study measured urinary creatinine^(29)^. This lack of quality assurance raises the possibility that the 24-h UIE values obtained from the collection of 24-h urine may be inaccurate.

Spot urine samples are preferable since they are simpler to collect, although iodide concentrations in spot urine samples are greatly variable during the day and throughout the seasons^(53)^. One study proposed that ten repeat spot urine collections should be utilised to evaluate a person’s iodine status with 20 % precision^(54)^. However, obtaining ten samples from an individual can be challenging. In this review, two studies used spot UIC, one collected spot samples before the collection of the 24-h urine^(35)^ and the other collected spot samples 2 h after breakfast^(31)^, and they did not comprehensively address the issue of variability in spot UIC over time. A study observed that the strongest correlation between UIC and 24-h UIE was derived from an afternoon spot urine sample^(55)^. This highlights the significance of selecting an appropriate time frame for spot urine collection in assessing iodine status. The studies described in this review highlight the potential of SIC monitoring for the assessment of iodine status at the population level, using median values as is currently adopted for spot urine samples. Multiple samples would be simpler for participants to collect, thereby diminishing the burden compared with spot urine sample, and would allow more precise estimation of salivary excretion than with single samples alone. It will be interesting to establish whether SIC proves to be an effective tool for monitoring risk of iodine deficiency in populations.

Goitre can be measured via thyroid ultrasonography or neck examination and palpation, and the rate of goitre can indicate long-term iodine status^(48)^. However, palpation of goitre has poor specificity and sensitivity in regions with a mild iodine deficiency^(56)^, and this may require the use of ultrasound to increase the accuracy of the diagnosis. Indeed, the use of ultrasound has been made possible even in distant and resource-constrained places, but the main drawbacks of this method are its cost, the demand for skilled technicians, and a significant degree of inter-user variability in its use and interpretation^(11)^. Other indicators that can be used to reflect iodine nutritional status are TSH and Tg^(48)^. TSH is a relatively insensitive indicator in adults^(1)^, while Tg has been shown to be more reliable when used to assess iodine consumption in children^(48)^. However, TSH and Tg measurements require blood sampling and processing with the additional challenge of needing to transport as frozen.

Finally, a major limitation of this scoping review is the low number of studies that met the inclusion criteria, and all the included studies differed in terms of their measurement methods and have some weaknesses; therefore, it will be necessary to conduct additional studies before a firm conclusion can be reached.

### Conclusion

The current scoping review has identified positive correlations between SIC and UIC and/or UIE, as well as between SIC and dietary iodine intake. The evidence reported suggests that the best time for obtaining saliva samples is either before or after dinner. In addition, the measurement of salivary iodide holds potential benefits for clinical practice, particularly in identifying iodine status among DTC patients undergoing a LID to assess the suitability for receiving ^131^I therapy. While SIC shows promise as a potential alternative biomarker for iodine status, the inadequate current data on saliva samples as a means of estimating iodide concentration for determining population iodine intake, along with significant variations among published study methods and populations, highlight the need for further investigations. More research is essential to establish a reference range for SIC as an iodine nutrition biomarker, determine the optimal timing and method for saliva sampling, assess the impact of various health conditions on SIC, and elucidate the role of iodide secretion in saliva.
